# EU-Trees4F, a dataset on the future distribution of European tree species

**DOI:** 10.1038/s41597-022-01128-5

**Published:** 2022-02-03

**Authors:** Achille Mauri, Marco Girardello, Giovanni Strona, Pieter S. A. Beck, Giovanni Forzieri, Giovanni Caudullo, Federica Manca, Alessandro Cescatti

**Affiliations:** 1 Faculty of Biological and Environmental Sciences, Organismal and Evolutionary Biology Research Programme, University of Helsinki, Helsinki, Finland; 2 European Commission, Joint Research Centre, Ispra, Italy

**Keywords:** Forestry, Climate-change ecology, Forestry, Forest ecology, Ecological modelling

## Abstract

We present “*EU-Trees4F*”, a dataset of current and future potential distributions of 67 tree species in Europe at 10 km spatial resolution. We provide both climatically suitable future areas of occupancy and the future distribution expected under a scenario of natural dispersal for two emission scenarios (RCP 4.5 and RCP 8.5) and three time steps (2035, 2065, and 2095). Also, we provide a version of the dataset where tree ranges are limited by future land use. These data-driven projections were made using an ensemble species distribution model calibrated using EU-Forest, a comprehensive dataset of tree species occurrences for Europe, and driven by seven bioclimatic parameters derived from EURO-CORDEX regional climate model simulations, and two soil parameters. “*EU-Trees4F*”, can benefit various research fields, including forestry, biodiversity, ecosystem services, and bio-economy. Possible applications include the calibration or benchmarking of dynamic vegetation models, or informing forest adaptation strategies based on assisted tree migration. Given the multiple European policy initiatives related to forests, this dataset represents a timely and valuable resource to support policymaking.

## Abstract (summary)

**Table:** 

Measurement(s)	trees occurences
Technology Type(s)	computational modeling technique
Factor Type(s)	trees • emission scenario • time step
Sample Characteristic - Organism	tree
Sample Characteristic - Environment	forest biome
Sample Characteristic - Location	Europe

Machine-accessible metadata file describing the reported data: 10.6084/m9.figshare.17144627

## Background & Summary

**Fig. 1:**
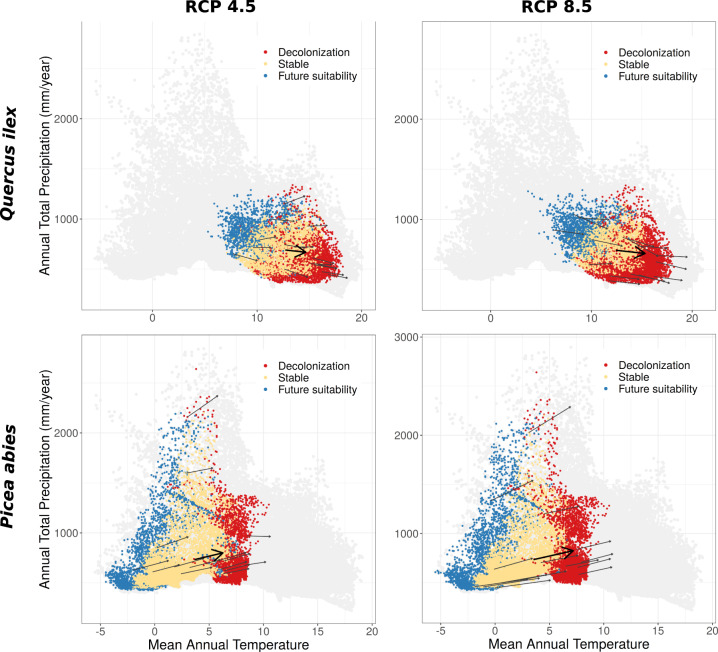
Two examples of the expected mismatch between the present climatic conditions in which tree species currently grow, and the climate they will experience by the end of the century under two contrasting warming scenarios (RCP 4.5 and RCP 8.5). Black arrows represent the variations in mean annual temperature and annual precipitation for fifteen random climate bins while the thick black arrow represents the average variations for the entire climate envelope of the species. Climate bins are shown with different colours: yellow, for stable areas where by the end of the century the tree species will still have a suitable climate; red, for decolonized areas where the tree species will no longer be climatically suited; blue, for future suitability areas that are not currently colonized by the tree species but will become suitable in the future. Grey dots show all combinations of mean annual temperature and total precipitation for the present and the future and for all 67 species studied here. The targeted species (*Quercus ilex* and *Picea abies*) were arbitrarily chosen from the full set of tree species.

Covering 35% of EU land^1^, forests play a fundamental economic and ecological role. Besides their obvious contribution to biodiversity and the provision of wood and non-wood products, forests maintain a wide range of ecosystem services, such as carbon storage and sequestration, habitat provision, and water regulation^2–4^. Nonetheless, forests are increasingly under threat from habitat fragmentation, the spread of invasive alien species, climate change, water scarcity, fires, storms, and pests^5,6^. By the end of the century, climate change alone will substantially alter the current distribution of climatically suitable areas for the majority of European trees species (Fig. 1), generating severe mismatches between species’ niches and the local climatic conditions^7–9^. This might result in both the erosion of current species ranges and colonization of newly suitable areas.

Considering the relatively low dispersal ability of most European tree species, it is unlikely that natural dispersion will permit forests to compensate for range erosion by colonizing new territory^8,10–12^. Targeted forest management appears an obvious and realistic option to minimize the loss of local biodiversity and ensure the continued delivery of forest ecosystem services^13,14^. This is particularly so in Europe, where forests are very far from naturalness;^15^ they have been managed for millennia through clearance to create croplands and pastures, and by intensive tree collection for fuelwood and construction materials^16,17^. Even now European forests are being managed rather intensively^1,18,19^. However, current management is mostly driven by economical considerations, while we strongly argue that ensuring a broad range of future forest ecosystem services in Europe urgently calls for a science-based change of direction to “design” forests capable to withstand environmental change while bringing economic, social and ecological benefits to humans and natural systems. Climate could change significantly in a given locality during the lifespan of an individual tree, therefore, forest management needs to consider not only the compatibility of target tree species with present climatic conditions but also with the climate expected in the near future^20–22^. Here we present a new dataset that, amongst others, might help forest managers to tackle these challenging issues. In particular, we provide current and future (to the end of the 21^st^ century) distribution maps for 67 tree species in Europe under different modelling and climatic assumptions.

The dataset has various features which make it stand out from available products. First, previous studies aiming at investigating the impact of climate change on tree species in Europe largely focused on few (<15) commercially important tree species^23–25^ (but see Thuiller *et al*.^26^ for a broader perspective). This might result in excluding tree species important for biodiversity, ecosystem functioning, wildlife habitat, and in turn ecosystem services. However, there is now increasing evidence that, in a climate change context, a greater number of plant species is vital to reduce vulnerability, guarantee ecosystem functioning and the future delivery of ecosystem services^27–29^. In fact, tree diversity is key to enhancing resilience of forest communities to climate-driven risks and disturbances, in particular when environmental conditions are rapidly changing^30,31^. Therefore, *EU-Trees4F* provides a more comprehensive view by mapping current and future ranges for a large number (67) of European tree species (Table S1).

Second, previous projections of future tree species ranges typically relied on bioclimatic parameters downscaled from coarse (grid cells greater than 100 km) simulations by one or more global climate models. Conversely, *EU-Trees4F* takes advantage of outputs of regional climate models (for Europe EURO-CORDEX)^32^ at a higher spatial resolution which have now become available.

Third, most previous studies focused on the potential distribution of tree species regardless of dispersal constraints, i.e. they provide suitable future areas of occupancy without making hypotheses about future colonization patterns^23–26,33–37^. Here, we provide both the potential future suitable areas of occupancy as limited only by climate and soil, and the expected future tree species distribution under the assumption of natural dispersal. In addition, we provide a version of the dataset where tree ranges are limited to future modelled land use^38^. Combined, these two pieces of information provide a useful tool to simulate management scenarios regarding the future of European forests. In fact, the potential suitable area set the climate boundaries for targeted forest management, while the natural dispersal scenario defines the likely trajectory in case of no human intervention on species distribution. The difference between the two scenarios can therefore be interpreted as the room for manoeuvre of forest adaptation strategies based on the assisted migration of tree species.

**Fig. 2:**
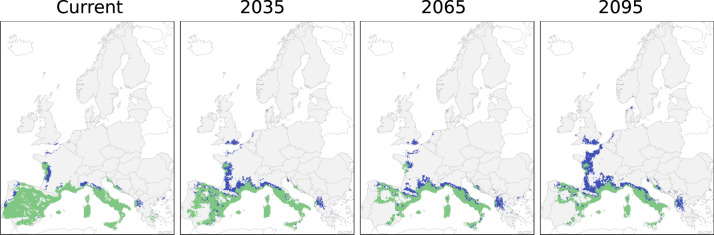
Comparison between the *Querus ilex* potential suitable range (according to ensemble SDM projections, green + blue) and the expected distribution as simulated using a dispersal model (Migclim, green) for an RCP 4.5 emission scenario. The blue area represents the potential suitable range that is not occupied by *Quercus ilex* due to dispersal limitations. The targeted species (*Quercus ilex*) was arbitrarily chosen from the full set of tree species.

The dataset includes future distribution maps corresponding to three 30-year periods, centred on 2035, 2065 and 2095, and modelled for two emission scenarios (RCP 4.5 and RCP 8.5) using an ensemble forecasting framework (Fig. 2 and Figure S1). Models were trained using the most comprehensive dataset of forest tree species occurrences in Europe currently available, EU-Forest^39,40^. Future projections were created using a set of 11 regional climate models (RCMs) from EURO-CORDEX^32^, downscaled to 5 arc-minutes (~10 km) spatial resolution.

This dataset has a wide array of potential applications in various research fields, including forestry, bio-economy, biodiversity and ecosystem services. We envisage that *EU-Trees4F* will facilitate active forest management for climate adaptation (including assisted colonization strategies)^41^ that addresses the balance between economic forest productivity (which currently hinges on rather few commercially exploited tree species) in the shorter or longer-term, the provision of non-economic ecosystem services, and the resilience of forest ecosystems to future environmental perturbations^22,30,31^. In addition, *EU-Trees4F* could contribute to biodiversity conservation and management to forecast changes in tree species richness through time^42^. In fact, the projected decline in tree species richness by the end of the 21^st^ century, might provide spatial information to foresters and practitioners concerning the areas that may require assisted recolonization^19^. Another possible application could be in forest pest management by providing spatial and temporal distribution of host tree species for harmful pathogens^43^. In addition, *EU-Trees4F* could serve as a benchmarking dataset to calibrate and/or evaluate the output from dynamic vegetation models^37^. Finally, our dataset could be used to support policy-making, given the current European Commission’s need to fulfil the European Green Deal’s^44^ objectives, the EU Biodiversity strategy 2030^45^, the EU Bio-Economy strategy^46^, and the new EU Forest Strategy^47^.

## Methods

**Fig. 3:**
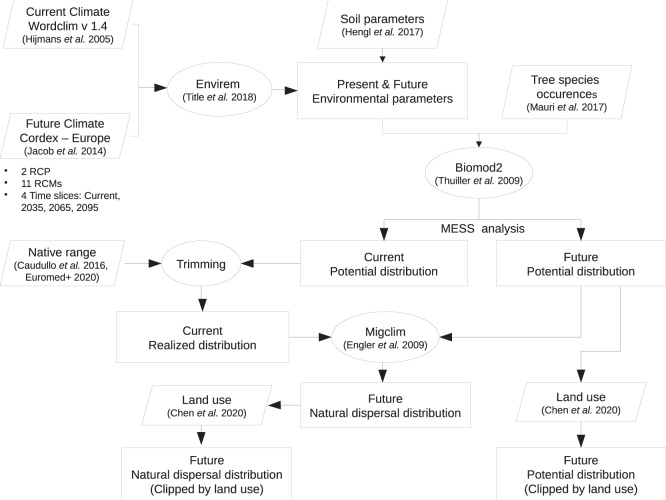
Flowchart illustrating the various steps undertaken to produce the distribution maps.

We produced a dataset of tree species distribution maps using a framework for species distribution modeling (SDM, BIOMOD2^48^), driven by a large database of tree species occurrences available at pan-European scale^39^. We included in our analysis 67 tree species that cover a broad range of life histories and climatic tolerances, ecosystem functions, ranging from the Mediterranean to the Boreal region (Table S1). We built an ensemble of tree species distribution models based on a set of nine environmental parameters describing key features of climate and soils (Table S2). We projected them into the future using 11 regional climate models from EURO-CORDEX, for two emission scenarios (RCP 4.5 and RCP 8.5), downscaled to a spatial resolution of 5 arc-minutes (Fig. 3). Additionally, we provided a version of the dataset masked by future land use^38^.

### Tree occurrences

**Fig. 4:**
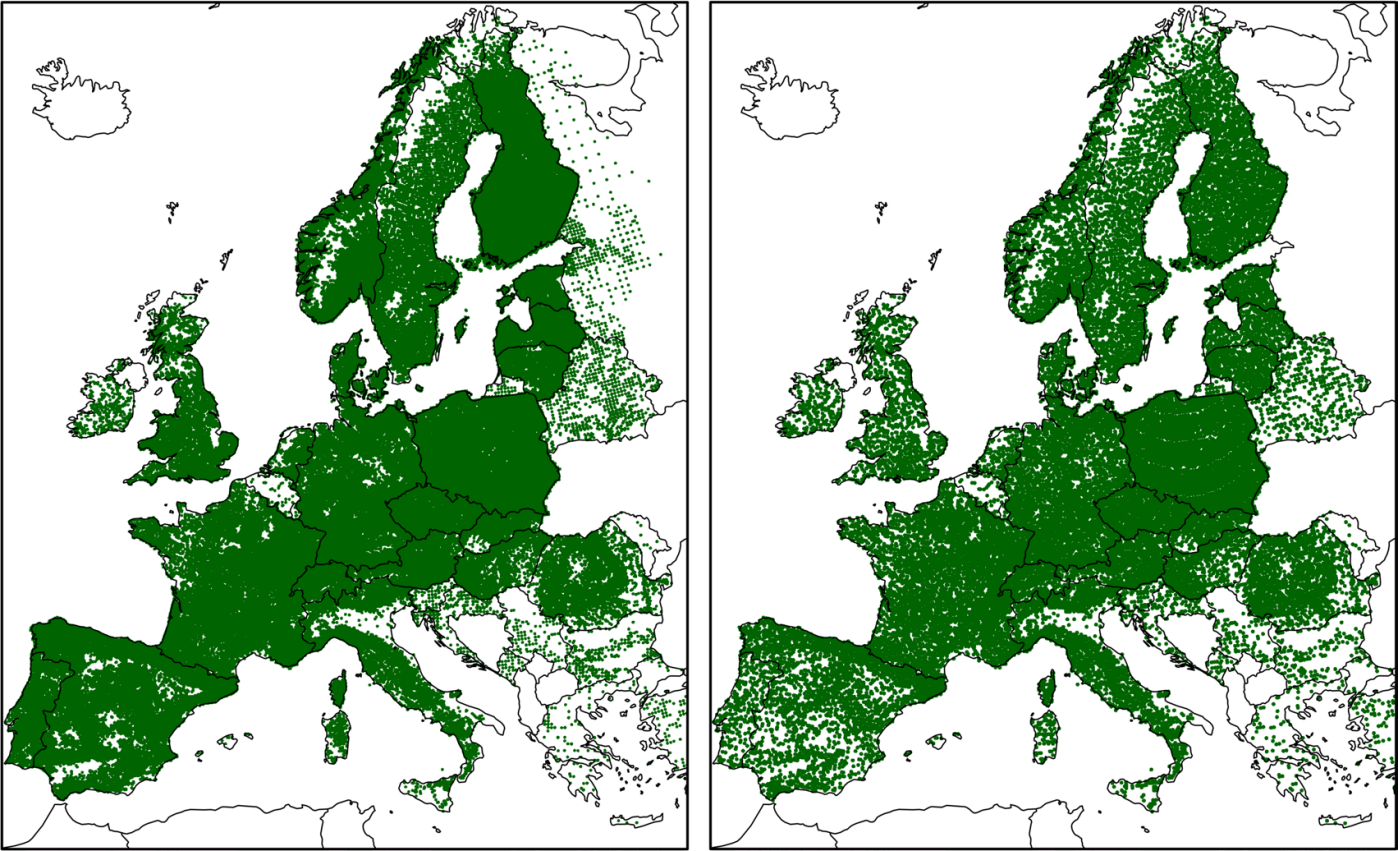
Spatial distribution of all the tree species occurrences (left panel) and the thinned occurrences used to train the species distribution models (right panel).

The core of our datasets is the EU-Forest data set that includes the best-quality data on forest tree species occurrences available in Europe^39,40^. We complemented EU-Forest with data from intensive monitoring plots (ICP Forests^49^) to fill in some geographical areas not well represented for a few species (Fig. 4). For Poland, which is an area with particular climate conditions not common elsewhere in our study domain, we enriched our dataset with occurrences obtained from Zając *et al*.^50^.

We considered as trees those species that have a defined crown and a single main stem, as defined in Gschwantner *et al*.^51^ for National Forest Inventory surveys in Europe. We included *Corylus avellana*, for its importance in terms of ecosystem functioning and services. As a result, we retain a set of 67 tree species totalling 582,066 occurrences. We note that this set covers only a portion of the entire pool of native tree species available in the study domain (443)^52^, but it includes all the tree species with significant commercial value as well as many tree species known for their importance for ecosystem functioning. However, some rare endemic tree species such as *Abies nebrodensis*, which is found in only a few forest patches in Sicily, are not included in our species list, while other tree species that are not commercially exploited but have a broader distribution (e.g. *Aria edulis*, *Pinus brutia, Prunus padus, Quercus coccifera, Sorbus aucuparia, Taxus baccata*) are included.

Prior to the analyses, we implemented a data thinning procedure to reduce spatial bias within the species occurrence records. First, we removed all the duplicates of the same tree species falling in each 5 arc-min (~10 km) cell. Second, similarly to Dyderski *et al*.^23^, we randomly selected a single occurrence in every 40 × 40 km grid cell and discarded the others. In this way, we overcame the problem of uneven sampling intensity in the occurrence datasets used^53^, and ensured that observations were evenly distributed within the geographical area of interest (Fig. 4). It is important to note that the environmental parameters used to develop the species distribution models with these thinned points describe the local surroundings (~ 10 km cell) rather than the ~ 40 km cells used for the thinning.

#### Environmental data

We selected climate and soil parameters considered critical to plant physiological functioning and survival^54–56^ and used in several earlier large-scale species distribution models^26,57–59^. These are winter and summer temperature (°C) and precipitation (mm/month), precipitation seasonality, mean annual temperature (°C), mean temperature of the coldest month (°C), total annual precipitation (mm/year), continentality, a humidity index (Alpha), growing degree days above 5 °C (GDD5), soil pH and organic carbon content (OCC) (Table S2). Four parameters (mean temperature of the coldest month, growing degree days above 5 °C, winter temperature, and the humidty index) were excluded as a result of multi collinearity tests made using the ‘usdm’ R package^60^ (Figure S2).

For the current period, the climatic parameters were derived from the Worldclim climatology Version 1.4^61^ with a spatial resolution of 5 arc-min (~10 km). It covers the period from 1961 to 1990, when climate conditions were more representative of the conditions experienced by the trees recorded in our occurrence dataset at the time of their establishment. GDD5, continentality and the humidity index (the ratio between actual and potential evapotranspiration) were calculated using the *envirem* library^62^.

For the future, we used 11 regional climate models simulations (RCMs) sourced from the Coordinated Regional Downscaling Experiment (CORDEX) of the World Climate Research Programme (WCRP). The EURO-CORDEX^32^ initiative, which is part of the CORDEX project, provides regional climate projections for Europe at ~12.5 km horizontal resolution (Table S3). These were downscaled to a 5 arc-minutes resolution (~10 km) using a change factor approach^63,64^, a widely used method in climate change impact assessments^65–69^. First, we computed monthly values from daily temperature and precipitation. From these, we calculated climate anomalies between the future and the control period, by applying a multiplicative correction for precipitation and an additive correction for temperature. The control period (1961–1990) is matching the period on which the Worldclim Version 1.4 climatology was computed. Climate anomalies were then gridded to a finer spatial resolution of 5 arc-minutes using bilinear interpolation, before adding them back to baseline Worldclim climatology. In addition, a climatic ensemble mean of the output of the 11 RCMs was calculated and used to project the species distribution into the future. We focused our analysis on two representative concentration pathways (RCP 4.5 and RCP 8.5) depicting the greenhouse gas concentration by the end of the 21^st^ century. The RCP 4.5 is an intermediate scenario corresponding to a projected change in global mean surface air temperature by the end of the century of + 1.8 °C relative to the reference period of 1986–2005^70^ and with greenhouse gases concentration stabilizing shortly after 2100. Instead, the RCP 8.5 is a business-as-usual scenario with steady increases in greenhouse gas concentrations resulting in a global mean surface air temperature increase of 3.7 °C by the end of the century relative to 1986–2005. Organic carbon content (g per kg) and soil pH, in the first 15 cm of topsoil, were extracted from the 1- km spatial resolution SoilGrids dataset^71^ and aggregated to a lower (5 arc-min) spatial resolution.

#### Species distribution modelling

**Fig. 5:**
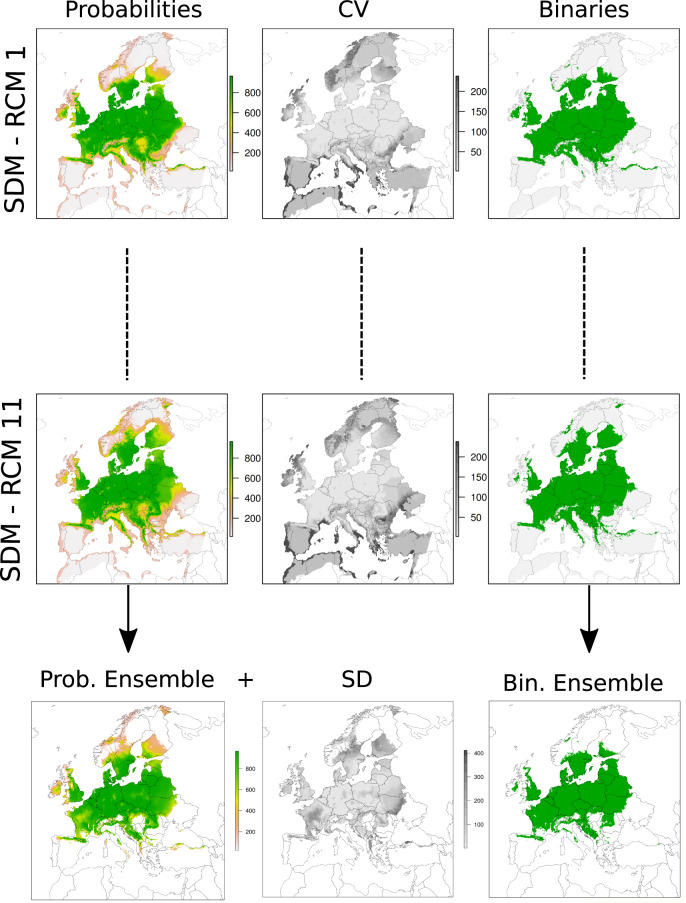
Illustrative example of the type of data produced in our modelling workflow. The results are shown for a single tree species *“Acer campestre”*, for a single time slice (2095) and for the emission scenario RCP 8.5. The upper and central panels show the output from BIOMOD2 run using the 11 regional climate models (RCMs). This includes the projection as probability maps (*Probabilities*) from the consensus model (average of the seven species distribution models implemented in BIOMOD2), the associated coefficient of variation (*CV*) among the seven SDMs, and the binary maps (*Binaries*). The lower panel shows the average value of the 11 consensus projections with associated standard deviation (*SD*) and ensemble binary map. In addition (not illustrated here), we ran BIOMOD2 using the average output of the 11 RCMs.

We modelled the potential distribution for each tree species using a well-accepted platform for ensemble species distribution modelling (BIOMOD2^48^) that is widely used for investigating the impact of climate change on forests^26,34,35,37,72^. We used six species distribution models: Generalized Linear Models (GLM), Generalized Additive Models (GAM), Generalized Boosting Models (GBM) or usually called Boosted Regression Trees, Multiple Adaptive Regression Splines (MARS), Maximum Entropy (Maxent) and Random Forest (RF). We used default settings except in MAXENT, where we sought to avoid overparameterization by setting product, threshold and hinge equal to false, as suggested in Merow *et al*.^73^. For each tree species, we calibrated the model on the thinned current occurrences and we predicted the potential distribution as a probability map, which values were converted for memory savings into integers ranging between zero and one thousand. Finally, for each tree species, we computed a consensus model by averaging the individual model’s projections (Fig. 5). Presences and absences were weighted equally by setting the prevalence parameter of BIOMOD2 to 0.5. We selected for each species 10,000 pseudoabsence points outside of its suitable area, as estimated from a surface range envelop model derived from the climatic predictors^74^. We projected the models (calibrated for current conditions) into the future using two emission scenarios (RCP 4.5 and RCP 8.5) and three 30-year time periods (centred on the years 2035, 2065, and 2095). Projections into current and future climates were made using two approaches: 1) A climatic ensemble mean that projects the consensus model into current and future conditions using the average output of the 11 RCMs, and 2) an ‘SDM’ ensemble mean that projects the consensus model for every single RCM, and a posteriori average of the outputs of the 11 SDMs. We calculated the ‘SDM’ ensemble mean for each grid cell, when at least 8 out of the 11 values were not missing data^75^ (Fig. 5). In addition, we translated probabilistic ensemble forecasts into binary maps using the same True Skill Statistics score as calculated for current predictions.

To the model projections in future climates, we applied the same 30-year time difference, as we had for the current projection, which was simulated with a climatology centred on the year 1975 but was referring to forest occurrences collected around the year 2005. The rationale behind this is that the tree occurrences in the calibration data set were recorded in the landscape around the year 2005 but mostly established themselves under the climatic conditions of the previous decades. Therefore, we assumed that the distribution of a species around the years 2035, 2065, and 2095 is predominantly constrained by the climatic conditions of the previous 30 years too.

In summary, for each tree species, we computed six SDMs and one consensus ensemble model. These were projected into current and future (three time periods) using two emission scenarios (RCP 4.5 and RCP 8.5). Future projections were made for 11 RCMs, either using a single climatic ensemble (average of the 11 RCMs) or by averaging the 11 consensus SDM output run using the single RCM output. Results produced from single climate model realization are provided in *EU-Trees4F* as well.

### Realistic dispersal scenario

**Fig. 6:**
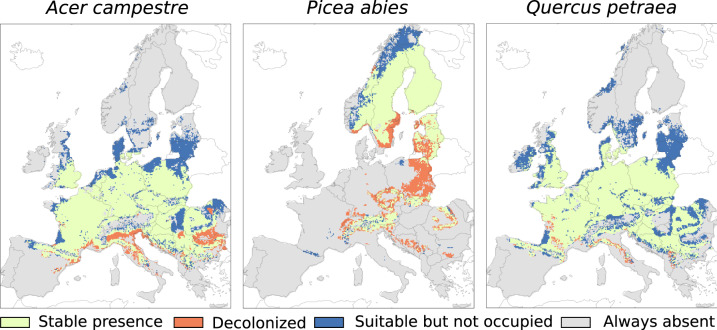
Illustrative example of Migclim output for three species modelled until the end of the century (2100) and for an emission scenario RCP 4.5. ‘Stable presence’ denotes areas that will remain suitable habitats from the present until the end of the century. ‘Decolonized’, indicates areas that will become climatically unsuitable by the end of the century. ‘Suitable but not occupied’, represents areas that will become climatically suitable by the end of the century but will not be naturally colonized due to dispersal limitations. The targeted species (*Acer Campestre, Picea abies* and *Quercus petraea*) were arbitrarily chosen from the full set of tree species.

We considered a realistic dispersal scenario that allows species to move from their initial position into climatically suitable areas according to their natural dispersal capacity (Fig. 6). We implemented the scenario using MigClim, a cellular automation dispersal model (MigClim^76,77^) that simulates dispersal, colonization, growth, and local extinction. MigClim used, for each tree species, the current and future binary distribution, as derived from BIOMOD2, for three time periods centred on the years 2035, 2065 and 2095 and a dispersal step of 30 years, which approximates the age at which most tree species become reproductive. As in Merow *et al*.^78^, we incorporated a spatial prior into our models, by trimming tree species occurrences by their native range distribution developed based on expert knowledge^79,80^. These constrained the predictions for the present in order to account for factors that were not included in the model covariates (e.g. biotic interactions or dispersal limitations).

Species dispersal was modelled using dispersal kernels driven by mean dispersal distances in combination with propagule production potential from the time a cell became colonized. We used a dispersal kernel based on a negative exponential function as implemented in other studies investigating plant species distribution in a changing climate^77,81–83^. Since species-specific mean dispersal distances are sparse in the literature, and not available for the entire set of tree species, we estimated them from maximum dispersal distances (MDD) using the following formula (Mean dispersal distance = 10 ^ (log10 MDD − 0.795)/0.984) derived from Tamme *et al*.^84^ and Thompson *et al*.^85^. We computed maximum distance dispersal using the dispeRsal algorithm (version 0.2)^84^ and for a few tree species we estimated it on the basis of authors’ knowledge of the species’ reproductive traits. The algorithm computes maximum dispersal distances using a linear mixed-effects model on the following functional traits downloaded from the TRY database^86^: species dispersal syndrome (in our case: wind, animal or no particular mechanism), growth form (tree), seed mass, realizing height (approximated to tree height) and terminal velocity when available (Table S1). Before the analysis, we log_10_ transformed seed mass, maximum dispersal distance and maximum plant height data. The maturity age was set to 1 since we were using a time step of 30 years, which is about the maturity age for most tree species. Propagule production was set to 1, which assumes that all mature trees will produce propagules. To assess variability due to randomness, for each species, we produced, and averaged, 30 replicates of the dispersal model.

### Land use datasets

We trimmed by land use the maps of future climatic suitability (potential occupancy) and the maps of future distribution expected under a scenario of natural dispersal. This was done for two emission scenarios (RCP 4.5 and RCP 8.5) and three time steps (2035, 2065, and 2095). We used a global land use dataset^38^ with a spatial resolution (3 arc-min), which was aggregated at the spatial resolution of our dataset (5 arc-min). We masked our dataset by the forest layer, which was derived by aggregating eight land use types as in Chen *et al*.^38^, specifically, needleleaf evergreen and deciduous trees, and broadleaf evergreen and deciduous trees from temperate, boreal and tropical regions. As these layers are presented as a cover fraction, we binarized them using a threshold of 40%, above which we consider to be forest. For the RCP 4.5 we used the SSP2 Socioeconomic Pathway scenario that represents a world where trends broadly follow their historical patterns with medium challenges to mitigation and adaptation (“a middle of the road”).

## Data Records

*EU-Trees4F* is available in GeoTIFF format at a resolution of 5 arc-minutes in LAEA (EPSG:3035) coordinate reference system. The files are freely accessible through *Figshare*^87^ (10.6084/m9.figshare.c.5525688) and https://forest.jrc.ec.europa.eu.

As illustrated in the main text and in Fig. 5, projections into future climates were made using two approaches: 1) A climatic ensemble mean that projects the consensus model from Biomod2 into future conditions using the average output of the 11 RCMs. These maps are stored in a binary format in the folder entitled “ens_clim”; 2) An ‘SDM’ ensemble mean that projects the consensus model for every single RCM, and a posteriori averages of the output of the 11 SDMs. These maps are stored in a binary format in the folder entitled “ens_sdms”. For the ‘SDM’ ensemble mean, we provided as well probability maps as GeoTIFF rasters in a WGS84 (EPSG:4326) reference system, together with the associated standard deviation calculated from the 11 consensus models for every single RCM.

In a third directory, entitled “single_models” we included GeoTIFF rasters in a WGS84 (EPSG:4326) reference system for the single climate species distribution model realizations. Here, there are three subdirectories: 1) “bin”, representing binaries distributions maps, 2) “prob” and 3) “CV”, representing respectively probabilities distribution maps and associated coefficient of variation maps. The coefficient of variation is relative to the six species distribution models implemented to produce the consensus projection from BIOMOD2. As an example, for a single tree species there are 66 GeoTIFF rasters maps in each subdirectory. These are based on 11 consensus SDM realizations (one for each RCM), two emission scenarios (RCP 4.5 and RCP 8.5), and three periods in the future (2035, 2065, 2095).

Finally, in the fourth folder entitled “datasets”, there are all the datasets needed to reproduce *EU-Trees4F*. In the “species occurrences” directory there are two subdirectories entitled “p_ICP”, which includes data from ICP-Forests, and “p_Poland” which includes additional occurrences from Poland. We merged these two datasets to the EU-Forest^39,40^ and we trimmed the merged occurrences by the species native ranges^79,80^ as described in the main text. The resulting dataset, in the subdirectory “input_sdm” was used as input to BIOMOD2 together with the environmental parameters included in the folder “climate” and “soil”.

## Technical validation

### Tree occurrences

We excluded from our analyses alien tree species, with the exception of *Robinia pseudoacacia* which is a highly naturalized tree species in Europe^88^. We further excluded tree species that had fewer than 30 occurrences as well as those occurrences located in areas that are climatically suitable for the species to survive but not to reproduce. For instance, *Quercus ilex* is present and surviving in Scotland because it has been planted there, but the climate doesn’t allow it to reproduce. To keep such cases from skewing our projections, we filtered out occurrences outside a species’ natural range as documented in chorological maps^79^. For the few species without detailed chorologies, we used the native distribution information taken from the Euro + Med PlantBase^80^ at the country level (accessed date: 11–11–2019). For *Robinia pseudoacacia*, we considered all the occurrences in the dataset. Overall we retained a set of 67 tree species totalling 589,862 occurrences. We further filtered the dataset by applying a thinning procedure aimed at removing spatial biases in the dataset. Altogether we retained 50,453 thinned occurrences that were used to train the species distribution model (Fig. 4).

### Environmental parameters assessment

**Fig. 7:**
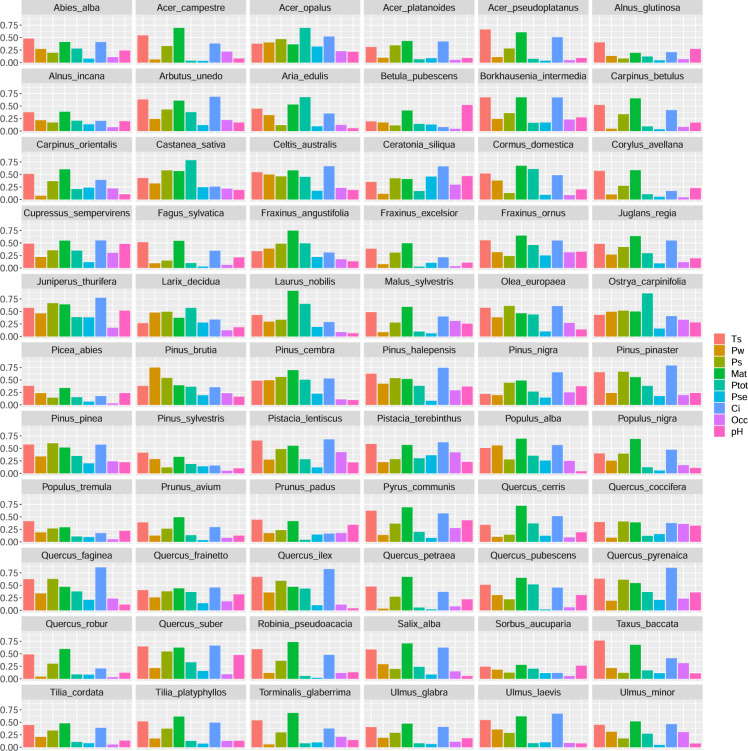
Relative importance of environmental parameters in the distribution models as calculated using the BIOMOD2 package. Ts = summer temperature; Pw = winter precipitation; Ps = summer precipitation; Mat = mean annual temperature; Ptot = total annual precipitation; Pse = precipitation seasonality; Ci = continentality; Occ = Organic carbon content; pH = soil pH.

We assessed the relative importance of individual environmental parameters to predict the distribution of each tree species. The assessment relied on a permutation procedure, as described in Thuiller *et al*.^48^. We calculated the Pearson correlation coefficient (*r*) between the model predictions and a prediction generated when the climatic variable in question was randomly permutated. We repeated the procedure ten times and kept the mean of all the *r* values. A high *r* (little difference between the two predictions) means that the permutated variable is not important for the model. Conversely, a low value reflects a significant difference between the predictions and therefore a high importance of the variable. The importance is therefore expressed as 1- *r*. Table 1 presents the relative importance of the individual environmental parameters averaged across the entire set of tree species, while Fig. 7 presents the results for all the species modelled.

**Table 1:** Relative importance of climatic and soil parameters averaged for the entire pool of tree species.

	Pse	Occ	pH	Pw	Ptot	Ps	Ci	Ts	Mat
Mean	0.13	0.17	0.21	0.25	0.28	0.35	0.46	0.49	0.55

Pse = precipitation seasonality; Occ = Organic carbon content; pH = soil pH; Pw = winter mean precipitation; Ptot = total annual precipitation (mm/year); Ps = summer mean precipitation; Ci = continentality; Ts = summer temperature (°C); Mat = mean annual temperature (°C).

### SDM evaluation and uncertainties

We evaluated the models’ predictive performances through a block-cross validation^89^, which is a particularly suited validation method to assess model transferability in geographic and environmental space where non-analog climatic conditions might be present^89^. Model validation results are reported using the true skill statistic^90^ (TSS), which takes into account both omission and commission errors, and, unlike other statistical measures such as KAPPA, is not affected by prevalence^90^. For each tree species, we performed the block-cross validation, and then averaged the results. Table 2 shows the results of model evaluation as an average by model for the entire pool of tree species. In addition, in Figure S3, we present the results for the entire pool of tree species.

**Table 2:** Overall model evaluation using the True Skill Statistics (TSS), averaged over the full set of tree species.

	GAM	GBM	GLM	MARS	ME	RF	MEAN
**TSS**	0.73	0.77	0.74	0.73	0.75	0.75	**0.74**

Generalized Additive Models (GAM); Generalized Boosting Models (GBM); Generalized Linear Models (GLM); Multiple Adaptive Regression Splines (MARS); Maximum Entropy (Maxent), Random Forest (RM); Mean of all models (MEAN).

In addition, we computed a consensus model by averaging single model predictions for each tree species. In doing so, we retained only predictions from models with TSS > 0.7, to avoid working with poorly calibrated models. We projected the consensus model into present and future climates and we computed the coefficient of variation (CV) from the single SDM projections. The higher (lower) the CV, the higher (lower) the uncertainty.

Finally, to avoid extrapolation to non-analog climates^91^, we excluded the areas of the projections where the future climatic conditions are unlike any currently observed in Europe. The exclusions were based on multivariate environmental similarity surface^92^ (MESS), which was created using the “mess” function from the “Dismo” package of the R library.

## Supplementary information

Supplementary Information

## Data Availability

Three scripts are available in figshare^87^. *“1_EU-Trees4F_sdm_present_future.R”* runs BIOMOD2 to model tree species distributions until the end of the century. It uses tree species occurrences and current/future environmental parameters that are stored in the “datasets” directory. This script does the following tasks: a) projects BIOMOD2 model output into the current and future environmental conditions. The projection into the future is made using two approaches: 1) A climatic ensemble mean of the 11 RCMs, or 2) an ‘SDM’ ensemble mean that projects the consensus model for every single RCM, and a posteriori averages the outputs of the 11 SDMs; b) calculates multivariate similarity between current and future projections, to avoid extrapolation to non-analog climates, c) derives the realized niche for each tree species by trimming the potential distribution maps with their native ranges distributions. The other two scripts calculate dispersal into the future using output from the first script. *“2_EU-Trees4F_dispersal_migclim_ens_clim.R”* using a climatic ensemble mean, whereas *“3_EU-Trees4F_dispersal_migclim_ens_sdms.R”* uses the ‘SDM’ ensemble mean. The directory “datasets” contains various files that are needed for the code to run, such as the environmental parameters and the species occurrences. All scripts were written and run in R software version 3.6.3^93^ (2020–02–29).
