# Author Correction: EU-Trees4F, a dataset on the future distribution of European tree species

**DOI:** 10.1038/s41597-023-01944-3

**Published:** 2023-01-19

**Authors:** Achille Mauri, Marco Girardello, Giovanni Strona, Pieter S. A. Beck, Giovanni Forzieri, Giovanni Caudullo, Federica Manca, Alessandro Cescatti

**Affiliations:** 1 Faculty of Biological and Environmental Sciences, Organismal and Evolutionary Biology Research Programme, University of Helsinki, Helsinki, Finland; 2 European Commission, Joint Research Centre, Ispra, Italy

**Keywords:** Forestry, Climate-change ecology, Forestry, Forest ecology, Ecological modelling

Correction to: *Scientific Data* 10.1038/s41597-022-01128-5, published online 3 February 2022.

In this article the acknowledgement of some data providers was omitted. The original article has been corrected.

