# Supplementary material for: EU-Trees4F, a dataset on the future distribution of European tree species
Source: Sci Data. 2022 Feb 3;9:37. doi: 10.1038/s41597-022-01128-5 (PMC8813948; doi:10.1038/s41597-022-01128-5)
Supplement: Supplementary file 1 — Supplementary Information [file 41597_2022_1128_MOESM1_ESM.docx]

### *Supplementary information to:* ***EU-Trees4F, a*** *dataset of the future distribution of European tree species*

***Supplementary material:***

***- Table S1***

***- Figure S1***

***- Table S2***

***- Figure S2***

***- Table S3***

***- Figure S3***

| **Species name** | **26** | **28** | **66** | **3106** | **MDD** |
| --- | --- | --- | --- | --- | --- |
| *Abies alba* | 70.5 | wind.special | *n.a.* | 68 | 800 |
| *Acer campestre* | 64.5 | wind.special | 1.1 | 25 | 175 |
| *Acer opalus* | 77.5 | wind.special | *n.a.* | 20 | 153 |
| *Acer platanoides* | 124.3 | wind.special | 1.0 | 35 | 482 |
| *Acer pseudoplatanus* | 90.6 | wind.special | 1.0 | 40 | 5000 |
| *Alnus glutinosa* | 1.8 | wind.special | 2.4 | 40 | 500 |
| *Alnus incana* | 1.2 | wind.special | *n.a.* | 30 | 494 |
| *Arbutus unedo* | 4.0 | animal | *n.a.* | 12 | 1761 |
| *Aria_edulis* | 61.7 | animal | *n.a.* | 25 | 949 |
| *Betula pendula* | 0.3 | wind.special | 0.8 | 30 | 475 |
| *Betula pubescens* | 0.2 | wind.special | 0.8 | 30 | 475 |
| *Borkhausenia_intermedia* | 78.7 | animal | *n.a.* | 15 | 727 |
| *Carpinus betulus* | 47.3 | wind.special | 1.2 | 30 | 425 |
| *Carpinus orientalis* | 14.8 | wind.special | *n.a.* | 20 | 232 |
| *Castanea sativa* | 2,692.8 | animal | *n.a.* | 35 | 480 |
| *Celtis australis* | 195.8 | animal | *n.a.* | 25 | 1777 |
| *Ceratonia siliqua* | 196.8 | animal | *n.a.* | 16 | 648 |
| *Corylus avellana* | 1192.3 | animal | *n.a.* | 6 | 15000 |
| *Cormus domestica* | 22.1 | animal | *n.a.* | 30 | 1296 |
| *Cupressus sempervirens* | 7.7 | wind.special | *n.a.* | 35 | 60 |
| *Fagus sylvatica* | 208.0 | animal | *n.a.* | 50 | 32 |
| *Fraxinus angustifolia* | 56.6 | wind.special | *n.a.* | 30 | 149 |
| *Fraxinus excelsior* | 60.2 | wind.special | *n.a.* | 43 | 725 |
| *Fraxinus ornus* | 29.9 | wind.special | *n.a.* | 20 | 146 |
| *Juglans regia* | 9,071.6 | animal | *n.a.* | 50 | 498 |
| *Juniperus thurifera* | 30.5 | animal | *n.a.* | 20 | 1381 |
| *Larix decidua* | 6.3 | wind.special | 0.8 | 56 | 171 |
| *Laurus nobilis* | 374.8 | animal | *n.a.* | 20 | 1777 |
| *Malus sylvestris* | 23.4 | animal | *n.a.* | 19 | 1060 |
| *Olea europaea* | 249.4 | animal | *n.a.* | 20 | 1331 |
| *Ostrya carpinifolia* | 10.6 | animal | *n.a.* | 20 | 250 |
| *Picea abies* | 6.4 | wind.special | 1.2 | 70 | 1500 |
| *Pinus brutia* | 43.6 | wind.special | *n.a.* | 35 | 376 |
| *Pinus cembra* | 256.5 | animal | *n.a.* | 50 | 12000 |
| *Pinus halepensis* | 20.6 | wind.special | *n.a.* | 27 | 402 |
| *Pinus nigra* | 20.1 | animal | *n.a.* | 50 | 7780 |
| *Pinus pinaster* | 54.8 | animal | *n.a.* | 40 | 5615 |
| *Pinus pinea* | 653.0 | animal | *n.a.* | 30 | 2814 |
| *Pinus sylvestris* | 7.9 | wind.special | 0.7 | 50 | 646 |
| *Pistacia lentiscus* | 20.8 | animal | *n.a.* | 8 | 1777 |
| *Pistacia terebinthus* | 37.8 | animal | *n.a.* | 10 | 1777 |
| *Populus alba* | 0.1 | wind.special | *n.a.* | 40 | 1043 |
| *Populus nigra* | 0.8 | wind.special | 0.3 | 58 | 789 |
| *Populus tremula* | 0.1 | animal | 0.1 | 40 | 15745 |
| *Prunus avium* | 183.5 | animal | *n.a.* | 40 | 2220 |
| *Prunus padus* | 91.4 | animal | *n.a.* | 19 | 774 |
| *Pyrus communis* | 31.0 | animal | *n.a.* | 30 | 1198 |
| *Quercus cerris* | 3,683.5 | animal | *n.a.* | 40 | 472 |
| *Quercus coccifera* | 1,856.6 | animal | *n.a.* | 12 | 337 |
| *Quercus faginea* | 1,805.7 | animal | *n.a.* | 25 | 1777 |
| *Quercus frainetto* | 2,309.1 | animal | *n.a.* | 40 | 1777 |
| *Quercus ilex* | 1,740.8 | animal | *n.a.* | 25 | 546 |
| *Quercus petraea* | 1,523.1 | animal | 6.5 | 45 | 300 |
| *Quercus pubescens* | 894.0 | animal | *n.a.* | 25 | 539 |
| *Quercus pyrenaica* | 1,305.7 | animal | *n.a.* | 30 | 532 |
| *Quercus robur* | 3,001.2 | animal | *n.a.* | 60 | 584 |
| *Quercus suber* | 3,621.9 | animal | *n.a.* | 25 | 546 |
| *Robinia pseudoacacia* | 19.0 | animal | 5.7 | 30 | 1441 |
| *Salix alba* | 0.1 | wind.special | *n.a.* | 35 | 984 |
| *Sorbus aucuparia* | 7.1 | animal | 4.1 | 27 | 1613 |
| *Taxus baccata* | 58.1 | animal | 5.6 | 29 | 1777 |
| *Tilia cordata* | 38.4 | wind.none | 1.3 | 43 | 373 |
| *Tilia platyphyllos* | 97.2 | wind.none | 1.7 | 40 | 373 |
| *Torminalis_glaberrima* | 74.8 | animal | *n.a.* | 33 | 907 |
| *Ulmus glabra* | 10.9 | wind.none | 0.5 | 48 | 350 |
| *Ulmus laevis* | 6.9 | wind.none | *n.a.* | 35 | 350 |
| Ulmus minor | 7.0 | wind.none | *n.a.* | 40 | 350 |

**Table S1.** Functional traits used to compute maximum distance dispersal (MDD) as described in Tamme *et al.* (2014)^1^ (see main text for more details). Column field names correpond to “Traits ids” as used in the TRY database^2^ from which the data were collected: 26: seed dry mass; 28: dispersal syndrome, 66: Seed terminal velocity; 3106: plant vegetative height.


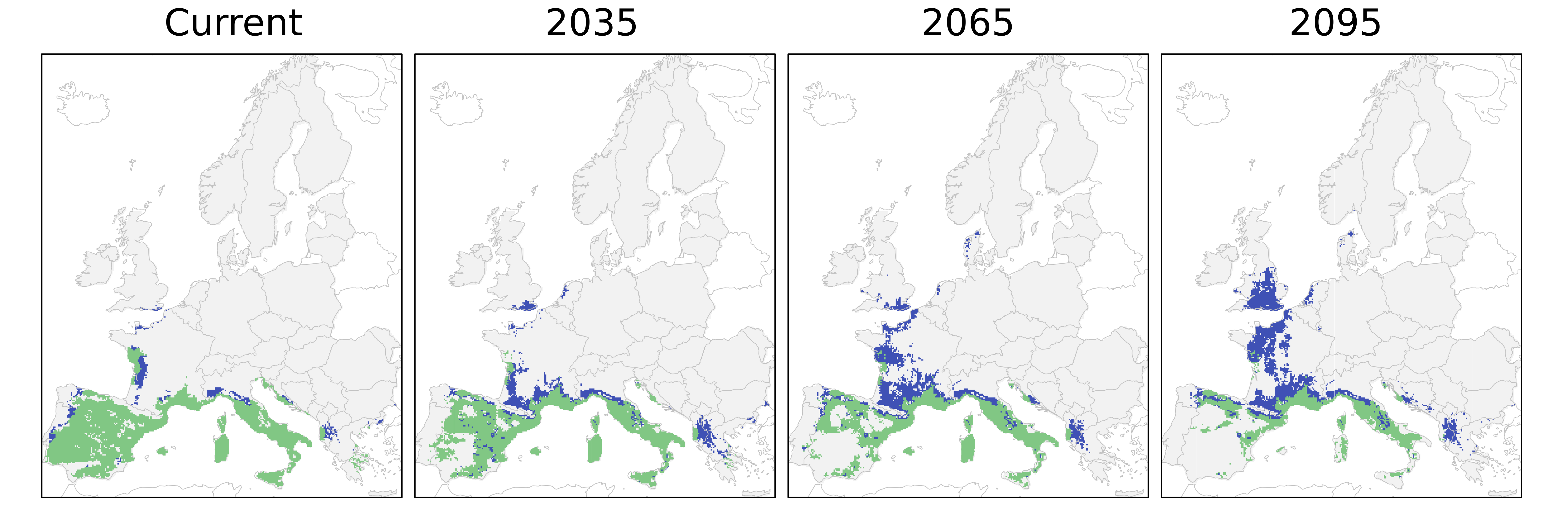
**Figure S1*.*** Comparison between the *Querus ilex* potential suitable range (according to ensemble SDM projections, green + blue) and the expected distribution as simulated using a dispersal model (Migclim, green). The blue area represents the potential suitable range that is not occupied by *Quercus ilex* due to dispersal limitations. The current distribution is modelled based on Worldclim V1.4 climatology, and future distributions based on EURO-CORDEX data for the scenario RCP 8.5. The targeted species (*Quercus ilex*) was arbitrarily chosen from the full set of tree species.

| **Climatic parameters** | **Meaning** |
| --- | --- |
| Winter Temperature (tw*) | Mean precipitation of December-January-February |
| Summer Temperature (ts) | Mean precipitation of June-July-August |
| Winter Precipitation (pw) | Sum precipitation of December-January-February |
| Summer Precipitation (ps) | Sum precipitation of June-July-August |
| Mean Annual Temperature (bio1) | Mean Annual Temperature |
| Mean Tempearature of the Coldest Month (bio6*) | Mean temperature of the coldest month of the year |
| Total Annual Precipitation (bio12) | Total Annual Precipitation |
| Precipiation seasonality (bio15) | Precipitaton seasonality (Coefficient of variation) |
| Continentality (ci) | Average temperature of the warmest month – average temperature of the coldest month |
| Climatic Moisture Index (Mi*) | This is the ratio between annual evaportraspiration and potential evapotranspiration |
| Growing Degree Days above 5°C (GDD5*) | Sum of the mean monthly temperature greater than 5°C, multiplied by the number of days |
| Soil pH (pH) | Soil pH measured in the top 15 cm of the soil |
| Organic Carbon Content (occ) | Organic Carbon Content measured in the top 15 cm of the soil |

**Table S2.** Climatic parameters used to drive the species distribution models. See methods for further details. The values with an astheriscs are the environmental parameters excluded after multi collinearity analysis (Figure S2).


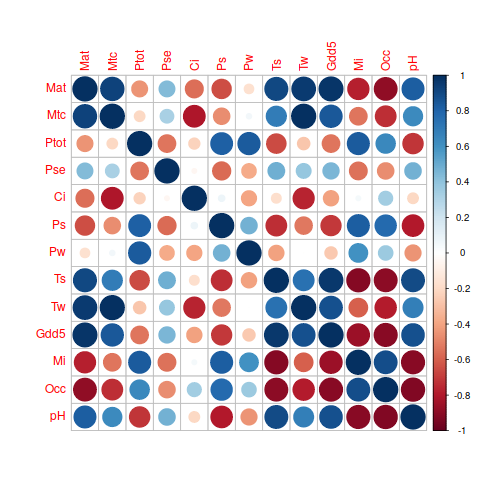


**Figure S2.** Correlation matrix showing the pairwise Spearman’s correlation coefficient among the environmental parameters considered in the study. Mat = Mean Annual Temperature, Mtc = Mean Tempearature of the Coldest Month*, Ptot = Total Annual Precipitation, Pse = Precipiation seasonality, Ci = Continentality, Ps = Summer precipitation, Pw = Winter precipitation, Ts = Summer temperature*, Tw = Winter temperature, Gdd5 = Growing Degree Days above 5°C*, Mi = Climatic Moisture Index*, Occ = Organic Carbon Content, Ph = Soil pH. The values with an astheriscs are the environmental parameters excluded after multi collinearity analysis.

| **Institute** | **RCM** | **Driving GCM** |
| --- | --- | --- |
| CLM-Community | CCLM4-8-17 | CNRM-CERFACS-CNRM-CM5 |
| CLM-Community | CCLM4-8-17 | ICHEC-EC-EARTH |
| CLM-Community | CCLM4-8-17 | MPI-M-MPI-ESM-LR |
| DMI | HIRHAM5 | ICHEC-EC-EARTH |
| IPSL-INERIS | WRF331F | IPSL-IPSL-CM5A-MR |
| KNMI | RACMO22E | ICHEC-EC-EARTH |
| SMHI | RCA4 | CNRM-CERFACS-CNRM-CM5 |
| SMHI | RCA4 | ICHEC-EC-EARTH |
| SMHI | RCA4 | IPSL-IPSL-CM5A-MR |
| SMHI | RCA4 | MOHC-HadGEM2-ES |
| SMHI | RCA4 | MPI-M-MPI-ESM-LR |

**Table S3.** Regional climate models (RCMs) used to project the species distribution model into the future. The driving Global Circulation Model (GCM) is indicated for each RCM in the right-hand column.

**
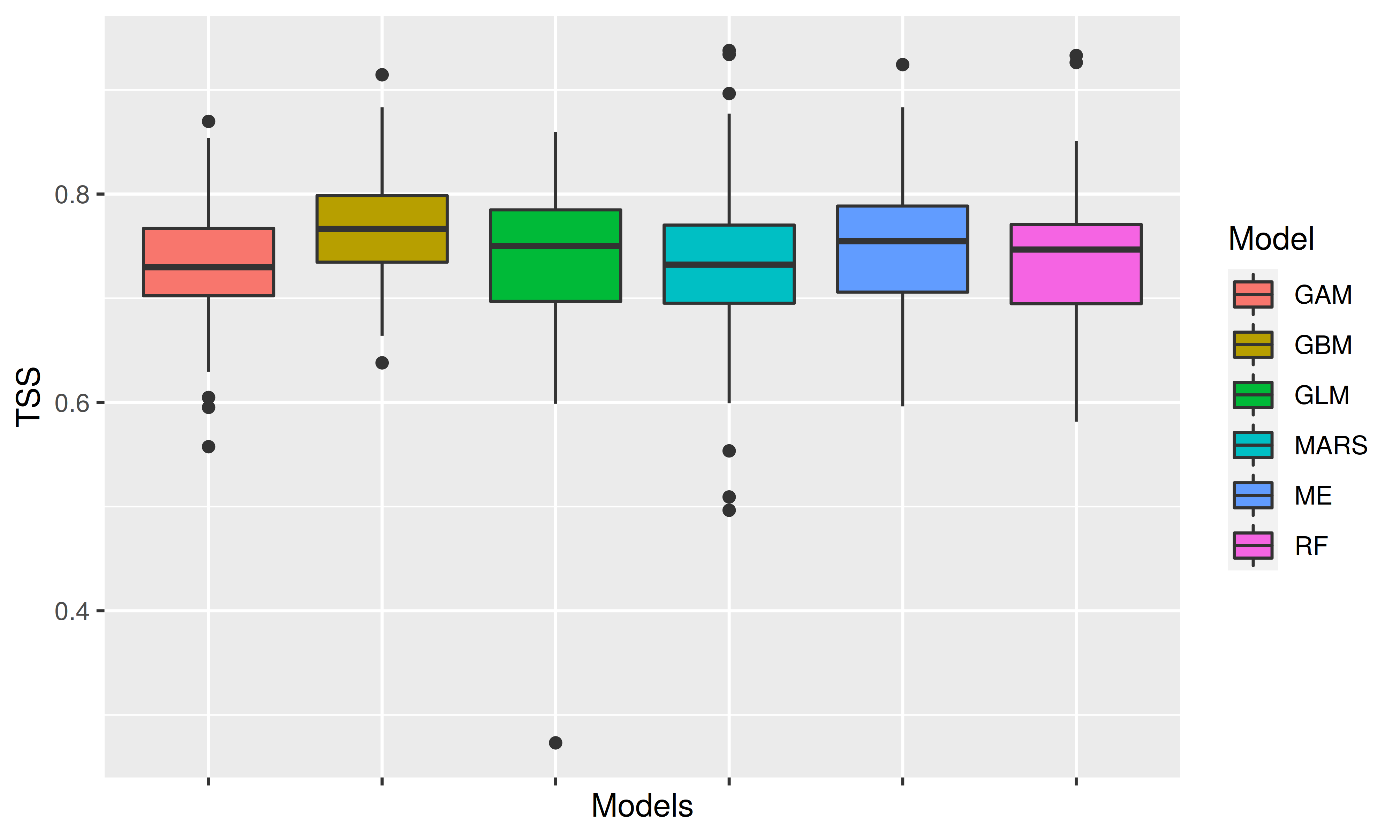
**

**Figure S3.** True skills statistics (TSS) scores for the species distribution models used to calculate the consensus ensemble projection: Generalized Additive Model (GAM), Generalized Boosting Model (GBM), Generalized Linear Models (GLM), Multiple Adaptive Regression Splines (MARS), Maximum Entropy - Maxent (ME), Random Forest (RF). Results are shown for the entire pool of tree species.

**References**

1. Tamme, R. *et al.* Predicting species’ maximum dispersal distances from simple plant traits. *Ecology* **95**, 505–513 (2014).

2. Kattge, J. *et al.* TRY plant trait database–enhanced coverage and open access. *Glob. Change Biol.* **26**, 119–188 (2020).
